# Biodesign and plant sciences: Evolving STEAM pedagogies in higher education – CORRIGENDUM

**DOI:** 10.1017/qpb.2025.10034

**Published:** 2025-12-17

**Authors:** Giovanna Danies, Carolina Obregón, Santiago Ojeda-Ramírez, Andrés Burbano

**Keywords:** biodesign, biomimicry, biotech education, design thinking, sustainable futures, corrigendum

The authors apologise for the author names having been published in the wrong order. They should have been listed as follows:

- Giovanna Danies
- Santiago Ojeda-Ramírez
- Carolina Obregón
- Andrés Urbano
  The authors sincerely apologise for any inconvenience this may have caused.
